# The role of toll-like receptors in acute and chronic lung inflammation

**DOI:** 10.1186/1476-9255-7-57

**Published:** 2010-11-25

**Authors:** Erin I Lafferty, Salman T Qureshi, Markus Schnare

**Affiliations:** 1 Division of Experimental Medicine, McGill University, Montréal, Québec H3A 1A3, Canada; 2 Department of Medicine, McGill University, Montréal, Québec H3A 1A1, Canada; 3 Institute of Immunology, Philipps-University of Marburg, Germany

## Abstract

By virtue of its direct contact with the environment, the lung is constantly challenged by infectious and non-infectious stimuli that necessitate a robust yet highly controlled host response coordinated by the innate and adaptive arms of the immune system. Mammalian Toll-like receptors (TLRs) function as crucial sentinels of microbial and non-infectious antigens throughout the respiratory tract and mediate host innate immunity. Selective induction of inflammatory responses to harmful environmental exposures and tolerance to innocuous antigens are required to maintain tissue homeostasis and integrity. Conversely, dysregulated innate immune responses manifest as sustained and self-perpetuating tissue damage rather than controlled tissue repair. In this article we review aspects of Toll-like receptor function that are relevant to the development of acute lung injury and chronic obstructive lung diseases as well as resistance to frequently associated microbial infections.

## Introduction

**Figure 1:**
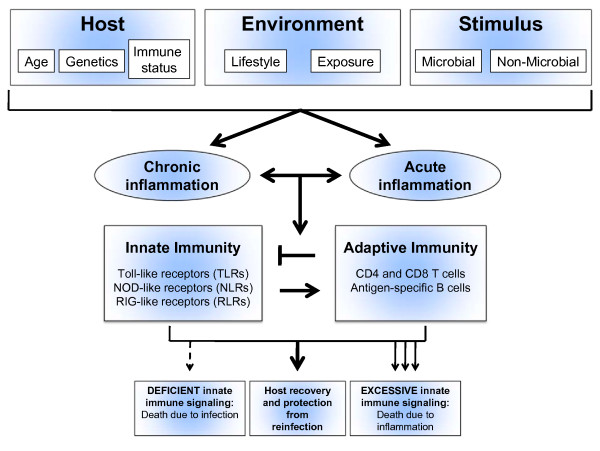
**Innate and adaptive immunity in acute and chronic lung inflammation**. A variety of host and environmental factors contribute to the development of acute and chronic lung inflammation. Recognition of pathogen associated molecular patterns (PAMPs) or endogenous damage associated molecular patterns (DAMPs) by host pattern recognition receptors (PRRs), including Toll-like receptors (TLRs), elicits innate immune responses that subsequently instruct adaptive immunity. Recovery from the inciting stimulus depends on robust yet tightly regulated innate and adaptive immune responses. Deficient innate immune signaling leads to excess pathogen burden while an exaggerated response can cause severe tissue injury and death of the host.

As an essential interface between the environment and the internal milieu, the lungs are continuously exposed to dust, pollen, chemicals, and microbial pathogens. Pneumonia and related patterns of lower respiratory tract infection are a frequent consequence of this interaction and account for a significant proportion of human morbidity and mortality throughout the world [1,2]. To contain potential environmental threats, the lungs are equipped with complex and multifaceted host defences. During tidal ventilation, the complex geometry of the nasal passages and branching pattern of the central airways impede the penetration of relatively large or heavy infectious particles while tight intercellular junctions ensure the structural integrity of the lung epithelium. This barrier is enhanced by airway goblet cells that secrete mucus and ciliated epithelial cells that constantly transport this viscous layer towards the bronchi and away from the alveoli to facilitate expulsion of trapped particles [3]. A variety of soluble host defence mediators such as secretory IgA, antimicrobial peptides, surfactant proteins, lactoferrin, and lysozyme also bolster the mucosal defences of the lower respiratory tract. Finally, resident alveolar macrophages (AMs) and airway mucosal dendritic cells (DCs) provide constant surveillance for potentially pathogenic factors while inhibiting T cell responses to myriad non-pathogenic antigens [4]. These normal host defences ensure that most encounters between the respiratory tract and pathogens are inconsequential; nevertheless, in response to prolonged, intense, or highly virulent microbial exposure, an inflammatory response or productive infection is likely to occur. To rapidly initiate an acute inflammatory response in these circumstances, the lung epithelium, myeloid cells, and associated lymphoid tissue are all equipped with a series of highly conserved pattern recognition receptor (PRRs) including Toll-like receptors (TLRs), NOD-like receptors (NLRs), and RIG-I like receptors (RLRs). PRR activation leads to the release of cytokines and chemokines that attract leukocytes to the site of infection and trigger the maturation and trafficking of antigen presenting cells for induction of adaptive immunity (figure 1). The purpose of this article is to review the role of TLRs in the pathogenesis or consequences of acute lung injury (ALI) and chronic inflammatory lung diseases including asthma, chronic obstructive pulmonary disease (COPD), and cystic fibrosis (CF).

## Ligands of TLRs

### Microbial ligands

Constant interactions between the respiratory tract and the environment pose a major challenge to host immunity and necessitate robust surveillance mechanisms to distinguish innocuous from pathogenic exposures. One strategy that is used by TLRs for selective induction of a host response is recognition of unique microbial structures termed pathogen-associated molecular patterns (PAMPs) [5-8]. Eleven functional TLR genes that play diverse roles in host defense, inflammation, autoimmunity, and neoplasia have been discovered in mouse and man (mouse TLR10 is a pseudogene and human TLR11 encodes a truncated protein) [5]. Prototypic examples of PAMPs include lipopolysaccharide (LPS), a outer membrane component of Gram-negative bacteria that stimulates TLR4 [8,9], bacterial lipoproteins that stimulate TLR2 in conjunction with either TLR1 or TLR6 [10], and flagellin, the protein monomer of bacterial flagella that activates TLR5 [11]. Nucleic acids are recognized by endosomal TLRs; double-stranded DNA with unmethylated CpG motifs activates TLR9 in a host species-specific manner while TLR3 and TLR7/8 are activated by dsRNA including synthetic poly (I:C) [12] and ssRNA, respectively [13,14].

### Host-derived ligands

Following the discovery that TLRs discriminate self from non-self through their intracellular localization or recognition of distinct ligand signatures, evidence was gathered in support of the hypothesis that endogenous host molecules termed danger associated molecular patterns (DAMPs) also stimulate TLRs. The first suggestion of this process came from studies of heat shock protein (hsp) [15]; subsequently, a number of other endogenous ligands including the extra domain A of fibronectin and hyaluronic acid were also shown to activate TLRs [16,17]. Recognition of endogenous ligands by TLRs may also contribute to the onset and initiation of autoimmune responses. For example, the high mobility group box protein 1 (HMGB1) protein that normally resides in the cell nucleus can activate TLR2 and induce hallmarks of lupus-like disease when released from apoptotic cells as a complex with nucleosomes [18].

## TLR signaling

The activation of TLRs results in acute inflammation and controls the adaptive immune response at various levels. Partially overlapping intracellular signaling pathways downstream of each TLR activate specific transcription factors that regulate the expression of genes responsible for inflammatory and immune responses. Four adaptors that harbour a Toll-Interleukin-1 Receptor (TIR) domain, including MyD88, TIRAP (MAL), TRIF (TICAM1), and TRAM, connect the TLRs to the cytoplasmic signaling machinery [5]. MyD88 was initially identified as part of the interleukin (IL) -1R and IL-18R signalling pathways and was subsequently implicated in signalling by almost all TLRs to trigger NF-κB, Interferon Regulatory Factor (IRF) 5, and Mitogen Activated Protein (MAP) kinase activation. A notable exception is TLR3 that mediates the activation of IRFs exclusively through the adaptor molecule TRIF [19]. The function of TIRAP is to recruit MyD88 to TLR2 and TLR4 at the plasma membrane, while TRAM recruits TRIF to TLR4 for activation of IRF3. A fifth adaptor protein, SARM, negatively regulates TRIF-dependent signaling [20,21]. Activation of different intracellular signaling mechanisms through TLRs results in the induction of distinct gene programs and cytokine expression patterns that control the recruitment of downstream molecules and regulate the identity, strength, and kinetics of gene and protein expression. More detailed reviews of the TLR signalling pathways have been published elsewhere [22-24].

The potent stimulatory responses mediated by TLR signaling must be tightly regulated at numerous levels in order to prevent the deleterious consequences of excessive innate immune activation [25]. For example, soluble forms of TLR4 and TLR2 may function as decoy receptors to terminate ligand interactions with membrane bound TLRs [26]. Furthermore, IRAK-M has 30-40% homology to the other IRAK-family members and stabilizes the TLR-MyD88-IRAK4 complex, leading to a unique negative regulatory influence on TLR signaling [27,28]. TLR signaling is also inhibited by transmembrane receptors like ST2, SIGIRR, and TRAILR while proteins such as Tollip [29], SARM [21], an inducible splice variant of MyD88 (MyD88s) [30], and the suppressor of cytokine signaling 1 (SOCS1) [31] are responsible for modulation of intracellular TLR signaling.

In addition to TLRs, a variety of other PRRs including the cytoplasmic NLRs and RLRs play important roles in the induction of lung inflammation. For example, the cytoplasmic NALP3 protein, a member of the NLR family that triggers assembly of the caspase-1 inflammasome and production of mature IL-1β, was recently implicated in the development of asbestos or silica-induced pulmonary fibrosis [32]. RLRs on immune and non-immune cells recognize viral RNA species and induce host responses through the adaptor IPS-1. Several putative cytosolic detectors of double-stranded DNA including DAI (ZBP1-DLM1) and AIM2 have also been identified; however their roles in lung diseases have not been established. A detailed discussion of these important non-TLR innate immune receptors is beyond the scope of this article; however, interested readers may consult other sources [33].

## Expression and function of TLRs in lung cells or tissue

TLRs are widely expressed on both resident lung cells as well as infiltrating cells of myeloid and lymphoid origin. Primary bronchial epithelial cells express mRNA for TLR1-10 and secrete the chemokine CXCL8 (IL-8) in response to various TLR ligands [34]. Human AMs have been shown to express low levels of TLR3, TLR5, and TLR9 and higher levels of TLR1, TLR2, TLR4, TLR7, and TLR8 [35,36]. Lung endothelial cells express TLR4 that is crucial for neutrophil recruitment and capillary sequestration following systemic LPS administration [37]. Neutrophils that localize to the lung vasculature in response to LPS express TLR1, TLR2, TLR4, TLR5, and TLR9 [38]. Several DC subsets have been identified in the mouse and human lung and can be distinguished according to their surface marker expression and anatomical location [39,40]. Lung DCs act as sentinels that are activated by TLR ligation in order to bridge innate and adaptive immunity. Lung plasmacytoid DCs (pDCs) express uniquely high levels of TLR7 and TLR9 that suppress the allergic response and regulatory lung DCs give rise to regulatory T cells [41]. Notably, in some cases the level of TLR transcription in cells does not correlate with functional responses [35,36]. For example, following stimulation with LPS or mycobacterial DNA, human AMs produced higher levels of the inflammatory cytokine TNF-α while interstitial macrophages produced higher levels of the immunoregulatory cytokines IL-6 and IL-10 despite similar levels of TLR mRNA [35]. Finally, lung tissue cells may also be activated through cooperative interactions with TLR responsive lymphoid cells, as exemplified by airway smooth muscle cell activation via IL-1β release from LPS-stimulated monocytes [42]. Thus, responsiveness to TLR ligands in the lung is shaped by cell intrinsic mechanisms as well as cooperative actions of both resident and recruited cell populations.

## Acute Lung Injury (ALI)/Acute Respiratory Distress Syndrome (ARDS)

ALI or ARDS is a life-threatening condition that is characterized by increased inflammatory cytokine expression and cell infiltration into the lungs, non-cardiogenic pulmonary edema, and diffuse alveolar damage that culminates in respiratory failure [43,44]. ALI can be a consequence of bacterial or viral infection or may be triggered by non-infectious insults including environmental toxin exposure (ozone, heavy metals), trauma, or hyperoxia. TLRs mediate ALI through recognition of microbial PAMPs or through detection of endogenous DAMPs (hsp, hyaluronan, fibrinogen, HMGB1 [16,45-50], both of which trigger inflammation [51-57]. Depending on the specific nature and intensity of the inciting stimulus, this response can be beneficial (maintenance of tissue integrity and repair) or detrimental (increased fibrosis and fluid in the lungs) for host recovery (figure 1) [43,57,58]. In this review we will focus on the contribution of TLR signaling to a subset of clinically relevant causes of ALI.

### Non-infectious causes of ALI/ARDS

Hemorrhagic shock (HS) is common in trauma patients and can prime the host immune response to elicit excessive inflammation, neutrophil influx and tissue injury in response to a secondary stimulus, causing ALI through the so-called 'two-hit hypothesis' [59-61]. Well characterized mouse models of HS-induced ALI using LPS as the secondary stimulus have determined that cross talk between TLR2 and TLR4 elicits heightened inflammatory mediator expression, such as CXCL1, leading to increased neutrophil influx and pulmonary edema [55,60,62-64]. Early inflammation in HS-induced ALI is dependent on upregulation of TLR4 by LPS, while later inflammation is mediated by heightened TLR2 expression on AMs and endothelial cells [64]. Deletion of either TLR2 or TLR4 in mice conferred protection from ALI and confirmed the presence of cross talk between these two receptors [63,65].

Hyperoxia (high concentrations of inspired oxygen) is a common therapy in critically ill patients; however, this treatment can also cause severe ALI by upregulating the production of reactive oxygen species [44,66-69]. TLR4 protects the host from hyperoxia-induced ALI by maintaining lung integrity and inducing the expression of protective anti-apoptotic factors such as Bcl2 and Phospho-Akt [70,71]. TLR4 or TLR2/TLR4 double knockout mice exposed to hyperoxia have significantly greater lung inflammation and permeability and are more susceptible to lethal ALI compared to wild type mice [71,72]. Conversely, TLR3-deficient mice are protected from ALI due to decreased neutrophil recruitment, induction of pro-apoptotic factors, and increased surfactant protein expression that clears injury-induced cellular debris [73-75].

Bleomycin is a potent anticancer agent that ultimately leads to cell death through generation of oxygen radicals and DNA breaks [76]. Bleomycin toxicity is usually associated with diffuse pulmonary fibrosis but may also cause ALI by triggering the degradation of high molecular weight hyaluronan (HA) in the extracellular matrix [77-79]. In contrast to intact HA that mediates homeostasis, accumulation of low molecular weight HA fragments is detrimental because it induces relentless pulmonary inflammation in AMs [72,78]. Loss of TLR2 and TLR4 or the adaptor molecule MyD88 leads to increased tissue injury, epithelial cell apoptosis and decreased survival following bleomycin exposure as well as decreased chemokine expression and defective neutrophil recruitment to the lungs [72]. Further mechanistic studies showed that TLR2 and TLR4 not only trigger basal NF-κB activation at the lung epithelium through interactions with intact HA in order to maintain cell integrity and decrease lung injury, but also mediate macrophage inflammatory responses to HA fragments following chemically induced tissue injury [72,80].

### Infectious causes of ALI/ARDS

Pneumonia is the most common cause of ALI or ARDS [81]. During the past decade, novel and highly virulent respiratory viruses, such as the Severe Acute Respiratory Syndrome Coronavirus (SARS CoV), have emerged as important causes of excessive lung damage in infected humans [82]. The 2003 global SARS epidemic had a 50% mortality rate with 16% of all infected individuals developing ALI [83,84]. The lung pathology in these patients mirrored ALI caused by other factors, consisting primarily of diffuse alveolar damage caused by virus-alveolar cell interaction [85]. The contribution of TLRs to SARS pathogenesis is not well understood [86]; however, using different mouse models of related CoV infection, a protective role for TLR4 [87] and MyD88 [88] has been suggested while TLR7 may be important for viral clearance through production of type I IFN [89].

Highly pathogenic strains of influenza virus are another important cause of ALI/ARDS in humans. Compared to seasonal influenza strains that bind cells of the upper respiratory tract, highly pathogenic H5N1 influenza virus infects alveolar type II cells, macrophages, and non-ciliated cuboidal epithelium of the terminal bronchi leading to a lower respiratory tract infection and ALI/ARDS [90,91]. Modeling of H5N1 infection in mice reproduced the pattern of damage seen in humans including increased neutrophilia, alveolar and interstitial edema, lung hemorrhage, and elevated TNF-α and IL-6 expression in the airway lining fluid [92,93]. Mice that survived beyond the acute phase of infection had large regions of lung interstitial and intra-alveolar fibrosis and ALI [93].

The role of TLRs has been intensively studied in influenza infection. TLR7 expression on pDCs plays a cell-specific role against influenza through MyD88-dependent IFN-α induction [13,94]. Despite the importance of TLR7/MyD88 signaling, MyD88-deficient mice can still produce type I IFN, control viral replication, and recover from the infection [95]. An increased lung viral load was seen only when MyD88 and IPS-1 (the adaptor molecule for the cytosolic RIG-I pathway) were both absent, suggesting that these pathways can compensate for one another during influenza infection [95]. Though not essential for survival, MyD88 does play a distinct role in the adaptive immune response to influenza through regulation of B-cell isotype switching [95,96].

The role of TLR3 in the immune response to influenza has been debated in the literature. Although several studies have shown that dsRNA is not produced during influenza replication [97,98], very low and potentially undetectable levels of this viral intermediate could still elicit a substantial immune response through TLR3 [99,100]. The finding that TLR3 is upregulated in human bronchial and alveolar epithelial cells during influenza infection suggests that it may play an important role in immune signaling [101]. Deletion of TLR3 leads to downregulation of inflammatory cytokine and chemokine production and an elevated viral load during the late phase of influenza infection. Surprisingly, TLR3 mutant mice have an increased survival rate compared to wild type mice suggesting that TLR3 signaling is detrimental to the host, despite its role in reducing viral replication [102,103]. In addition to the TLRs, RIG-I, NLRP3, and NOD2 have also been implicated in the immune response to influenza [104-108]; however, the relative contribution of these PRRs to influenza-specific host defense requires additional study.

## TLRs in chronic pulmonary diseases

### Cystic Fibrosis (CF)

CF is an autosomal recessive disorder caused by mutations in the cystic fibrosis transmembrane conductance regulator (CFTR) gene [109]. The airways of CF patients are characterized by chronic bacterial colonization and associated neutrophilic inflammation. *P. aeruginosa *infection is the major cause of morbidity and mortality among CF-affected individuals, producing acute pneumonia or chronic lung disease with periodic acute exacerbations [3,110,111]. A predisposition to chronic and progressive *P. aeruginosa *infection occurs despite the finding that both CF and non-CF lung epithelial cells express functional TLRs that can mediate inflammatory responses to microbes. For example, in one study comparing human CFTE29o (trachea; homozygous for the delta F508 CFTR mutation) and 16HBE14o (bronchial non-CF) cells, comparable mRNA and surface protein expression of TLR1-5 and TLR9 was observed [112]. TLR6 mRNA, but not protein, expression was observed in both cell lines; however, for unclear reasons only the CF line responded to TLR2/TLR6 agonist MALP-2 [112]. Despite this similar TLR expression pattern, a more recent study showed increased inflammatory responses following stimulation with clinical *Pseudomonas *isolates in a CF airway epithelial cell line (IB3-1) compared to a "CF-corrected" line stably expressing wild type CFTR [113]. A detailed analysis showed that these responses were dependent on bacterial flagellin and TLR5 expression. Peripheral blood mononuclear cells from CF patients also responded more vigorously to stimulation with *P. aeruginosa *and TLR ligands compared to healthy controls and expressed higher levels of TLR5 mRNA, suggesting that CFTR mutations modulate the host inflammatory response through undetermined mechanisms [113]. In another study, a selective increase in TLR5 expression was found on airway, but not circulating, neutrophils from CF patients compared to patients with bronchiectasis and healthy control subjects [38]. The functional relevance of neutrophil TLR5 expression was reflected by its correlation with lung function values in *P. aeruginosa*-infected CF patients. Neutrophils also had increased flagellin dependent IL-8 secretion, phagocytosis, and respiratory burst activity that were attributed to chronic infection rather than as a primary consequence of mutant CFTR [38]. TLR5-deficient mice showed impaired bacterial clearance, reduced airway neutrophil recruitment and MCP-1 production after low dose challenge with flagellated *P. aeruginosa *that was not observed after challenge with an isotypic non-flagellated strain, confirming a specific contribution of TLR5-dependent pathways to the host inflammatory response [114].

In addition to TLR5-dependent recognition of flagellin, *P. aeruginosa *LPS is detected by TLR4 and the *P. aeruginosa *ExoS toxin is recognized by both TLR2 and TLR4 [11,115-117]. Loss of a single TLR does not confer susceptibility to *P. aeruginosa *infection while deletion of the adaptor molecule MyD88 does confer hypersusceptibility, increased lung bacterial load, and deficient neutrophil recruitment [114,117-123]. Interestingly, MyD88 may play an essential role only during the early phase of infection (4-8 hours) as inflammation and control of bacterial load 48 hours after low dose infection occurred through an undetermined MyD88-independent mechanism [119]. Both TLR2 and TLR4 signal through MyD88-dependent and -independent pathways while TLR5 signals exclusively through MyD88. Studies to determine the relative contribution of TLR2, TLR4, and TLR5 have had conflicting results, possibly due to the complex pathogenesis of pseudomonal infection [123-125].

*Staphylococcus aureus *and *Burkholderia cenocepacia *have been associated with early and advanced CF lung disease, respectively [3]. *B. cenocepacia *provokes lung epithelial damage and TNF-α secretion that leads to severe pneumonia and sepsis in CF patients [126,127]. Excess inflammation and mortality in *B. cenocepacia *infection occurred through flagellin-dependent activation of TLR5 and MyD88 [128,129]. Another study showed that, despite higher bacterial load, MyD88-deficient mice had less inflammation and decreased mortality compared to wild type mice infected with *B. cenocepacia *[130].

### Chronic Obstructive Pulmonary Disease (COPD)

COPD includes disorders of the respiratory system that are characterized by abnormal inflammation as well as expiratory airflow limitation that is not fully reversible. In humans, the main risk factor for COPD is smoking and the disease prevalence rises with age [131]. Although the pathogenesis of COPD is not well understood, various aspects of lung innate immunity are impaired including mucociliary clearance, AM function, and expression of airway antimicrobial polypeptides [132]. As a result, microbial pathogens frequently establish residence in the lower respiratory tract and induce a vicious circle of inflammation and infection that may contribute to progressive loss of lung function [133] (figure 1).

There is accumulating evidence that impaired innate immunity is likely to contribute to the pathogenesis of COPD [134]. An essential role for TLRs in the maintenance of lung structural homeostasis under ambient conditions was recently described [135]. In this study, TLR4- and MyD88-deficient mice developed spontaneous age-related emphysema that was associated with increased oxidant stress, cell death, and elastolytic activity. A detailed mechanistic analysis showed that TLR4 maintains a critical oxidant/antioxidant balance in the lung by modulating the expression and activity of NADPH oxidase 3 in structural cells. In light of this finding, the free radicals and oxidant properties of tobacco smoke have been hypothesized to subvert innate immunity and cause lung cell necrosis and tissue damage [136,137]. Indeed, mice with short-term cigarette smoke exposure develop neutrophilic airway inflammation that is dependent on TLR4, MyD88, and IL-1R1 signaling [138]. Consistent with these findings, C3H/HeJ mice that have naturally defective TLR4 signaling develop less chronic inflammation after 5 weeks of cigarette smoke exposure [139]. Finally, long-term cigarette smoke exposure induced strain-dependent emphysema in mice in one study, although no specific association to TLRs was described [140].

Several studies have evaluated TLR expression and function in AMs from COPD patients, smokers, and non-smokers. Using flow cytometry, one group showed reduced TLR2 expression on AMs of COPD patients and smokers compared to non-smokers following *ex vivo *ligand stimulation. Upregulation of TLR2 mRNA and protein expression was observed only in AMs from non-smokers while no significant differences in TLR4 expression were demonstrated among these three groups [141]. Another report showed comparable AM expression of TLR2, TLR4 or the co-receptors MD-2 or CD14 between smokers and non-smokers [142], yet AM stimulation with TLR2 or TLR4 ligands elicited lower mRNA and protein expression of inflammatory cytokines (TNF-α, IL-1β, IL-6) or chemokines (IL-8, RANTES) in smokers that was associated with suppressed IRAK-1 and p38 phosphorylation and impaired NF-κB activation [142]. From this data, the authors concluded that chronic LPS exposure via cigarette smoking selectively reprograms AMs and alters the inflammatory response to TLR2 and TLR4 ligands [142]. Finally, another study showed reduced TLR4 mRNA expression in nasal and tracheal epithelial cells of smokers compared to healthy non-smoking control subjects with no differences in TLR2 expression [143]. Relative to non-smokers, patients with mild or moderate COPD showed increased expression of TLR4 and HBD-2, a TLR4 inducible antimicrobial peptide, while those with advanced COPD had a reduction in TLR4 and HBD-2 expression [143]. Modulation of TLR4 expression by cigarette smoke extract was studied *in vitro *and revealed a dose-dependent reduction in TLR4 mRNA and protein expression as well as reduced IL-8 secretion in the A549 alveolar epithelial cells [143]. Taken together, these findings point to dynamic regulation of airway epithelial and AM TLRs in response to diverse environmental stimuli. The differences in TLR expression across studies could be related to variable LPS content in tobacco smoke, bacterial colonization, or a persistent inflammatory state. Increased TLR4 expression in mild or moderate COPD may reflect a robust host response, while the decreased TLR4 expression level in association with severe COPD may reflect a loss of innate immunity or an adaptive regulatory response.

The interaction of cigarette smoke and PRR activation has been studied using mouse models. In one study, AMs from mice that had been exposed to cigarette smoke for eight weeks showed decreased cytokine (TNF-α, IL-6) and chemokine (RANTES) production following *in vitro *stimulation with double-stranded RNA, LPS, or NLR agonists [144]. No alteration of TLR3 or TLR4 expression was observed; however, there was decreased nuclear translocation of the transcription factor NF-κB. The functional impairment of cytokine release was specific to AMs and reversible after cessation of smoke exposure [144]. A subsequent report found a synergistic interaction of cigarette smoke and dsRNA or influenza virus that leads to emphysema in mice through epithelial and endothelial cell apoptosis as well as proteolysis [145]. This process was mediated by IL-12, IL-18, and IFN-γ as well as activation of antiviral response pathways including the intracellular signaling adaptor protein IPS-1 and the kinase PKR.

Defective innate immunity may predispose to acute exacerbations of COPD that are characterized by acutely worsening dyspnea, cough, sputum production, and accelerated airflow obstruction [146]. Bacterial colonization (*Streptococcus pneumoniae, Haemophilus influenzae*) or viral infection (Influenza A and B, Respiratory Syncytial Virus) of the lower respiratory tract are primary causes of acute COPD exacerbations [146-152]. Virulent pneumococci express the toxin pneumolysin that is able to physically interact with TLR4 [153-159]. Consistent with this finding, nasopharyngeal infection of TLR4-deficient mice with *S. pneumoniae *causes enhanced bacterial load, dissemination, and death compared to wild type mice [158]. Interestingly, the role of TLR4 seems to be specific to the nasopharynx as TLR4-deficient mice exhibit only a modest impairment of host defense following direct pneumococcal infection of the lower respiratory tract [160]. TLR2 is also upregulated following pneumococcal infection and enhances host inflammatory responses [161,162]. Despite a modest reduction of inflammatory mediator production, TLR2-deficient mice can still clear high and low infectious doses of *S. pneumoniae*, suggesting that another PRR compensates for the loss of TLR2 in this model [160,163]. TLR9-deficient mice are slightly more susceptible to pneumococcal infection compared to wild type animals [164]. Conversely, abrogation of MyD88 signaling leads to uncontrolled airway pneumococcal growth, systemic bacterial dissemination and decreased immune mediator (TNF-α and IL-6) expression [158,165,166]. The severe susceptibility phenotype of MyD88-deficient mice compared to mice with a single deletion of TLR9 or combined deletion of TLR2 and TLR4 highlights the crucial role of this downstream adaptor in host defense against *S. pneumoniae *[158,160,163,164,167].

Non-typeable *H. influenzae *(NTHi) is another bacterium that commonly colonizes the respiratory epithelium and causes COPD exacerbations [168-171]. While NTHi produces both TLR4 and TLR2 ligands, TLR4/MyD88 is the dominant immune signaling pathway *in vitro *and mediates bacterial clearance *in vivo *[172]. TLR4 signaling in response to NTHi is entirely MyD88 dependent as TRIF KO mice had an identical bacterial load compared to wild type mice [172]. TLR3 may also play a role in inflammatory mediator production in the immune response to NTHi although its relative contribution to bacterial clearance is not clear [173].

### Asthma

Asthma is a potentially life-threatening chronic inflammatory airway disease that is characterized by episodic bronchoconstriction, mucus hypersecretion, goblet cell hyperplasia and tissue remodelling that may begin in childhood. The underlying immune response in asthma is targeted against environmental antigens including pollen or dust particles and is characterized by the presence of antigen-specific Th2 cells in the lung that facilitate production of antigen specific IgE [174,175]. Viral and bacterial infections have been associated with induction or protection against asthma, suggesting that innate immunity plays an important role in disease pathogenesis [176]. On the basis of several epidemiologic, human, and animal studies, the timing and extent of LPS exposure, and presumably TLR4 activation, appears to determine whether a protective Th1 response or a permissive Th2 response develops in the lung [177]. For example, it was demonstrated that low dose administration of intranasal LPS induces a Th2 biased immune response in the lung whereas elsewhere in the body LPS is a strong inducer of a Th1 immune response [178]. Nevertheless, experimental treatment of mice with microbes [179] or TLR agonists [180,181] inhibits allergic sensitization, eosinophilic inflammation, and airways hyperresponsiveness. Recently, experimental intranasal infection of pregnant mice with *Acinetobacter lwoffii *F78 was shown to confer protection against ovalbumin-induced asthma in the progeny. Using knockout mice, the protective effect was shown to be dependent on maternal TLR expression and suggests that microbial recognition during pregnancy somehow primes the fetal lung environment for a Th1 response later in life [182].

Lung resident cells that express TLR4 also play an important role in the induction of allergen specific Th2 cells via recognition of house dust mite (a ubiquitous indoor allergen) that leads to the production of thymic stromal lymphopoietin, granulocyte-macrophage colony-stimulating factor, IL-25 and IL-33. This cytokine milieu can bias lung DCs towards a Th2 activating phenotype that drives the polarization of naïve lymphocytes [183]. In addition, eosinophil derived neurotoxin can induce TLR2-dependent DC maturation, leading to Th2 polarization by secretion of high amounts of IL-6 and IL-10 [184] while basophils may also instruct T cells to become Th2 cells [185].

TLRs have been shown via genetic association studies as well as single and multiple gene knockout studies to play a role in the development of allergic asthma. For example TLR7 and TLR8 are associated with human asthma [186] while ligands of TLR7 and TLR8 can prevent airway remodeling caused by experimentally induced asthma [187,188]. TLR10 single nucleotide polymorphisms have also been associated with asthma in two independent samples [189] although the ligand for TLR10 has not been defined. Finally, in a multi-centre asthma study, TLR4 and TLR9 were both associated with wheezing and TLR4 was also associated with allergen specific IgE secretion [190]. Based on this observation, TLR9 ligands are currently in clinical trials for the treatment or prevention of asthma [191].

Asthma can be further exacerbated by bacterial respiratory tract infection including *Mycoplasma pneumoniae *or *Chlamydophila pneumoniae *[192]. In one study, 50% of patients suffering from their first asthmatic episode were infected with *M. pneumoniae *while 10% were serologically positive for acute *C. pneumoniae *infection [193,194]. MyD88-deficient mice infected with *C. pneumoniae *failed to upregulate cytokine and chemokine expression, had delayed CD8^+ ^and CD4^+ ^T cell recruitment, and could not clear the bacterium from the lungs leading to severe chronic infection and significantly increased mortality [195]. At a later stage of infection, IL-1β, IFN-γ and other inflammatory mediators may be upregulated via a MyD88-independent pathway but are not sufficient to prevent mortality from *C. pneumoniae *[195]. TLR2 and TLR4-deficient mice can recover from *C. pneumoniae *infection with no impairment of bacterial clearance suggesting that other PRRs are also involved in host defense or that TLR2/TLR4 act in concert during *C. pneumoniae *infection [195,196].

TLR2 is also upregulated in response to *M. pneumoniae *infection, leading to increased expression of airway mucin [197,198]. Allergic inflammation along with the induction of Th2 cytokines (IL-4, IL-13) leads to TLR2 inhibition during *M. pneumoniae *infection, thereby decreasing the production of IL-6 and other Th1 proinflammatory mediators that are required for bacterial clearance [199]. Antibiotic treatment of asthmatic patients infected with *M. pneumoniae *improves their pulmonary function and highlights the increasingly important role that bacterial colonization and interactions with the host innate immune response play in asthma exacerbations and mortality [200,201].

Viral infection of the lower respiratory tract can also contribute to asthma development and exacerbations. Respiratory Syncytial Virus (RSV) is a particularly important cause of acute bronchiolitis and wheezing in children that may lead to the subsequent development of asthma [202-206]. Wheezing after the acquisition of severe RSV infection early in life has been associated with elevated Th2 responses, eosinophilia, and IL-10 production [207-211]. During RSV infection, the viral G protein mediates attachment to lung epithelial cells and the F protein leads to the fusion of the viral envelope with the host cell plasma membrane [212]. In response to RSV infection, TLRs are broadly upregulated in the human tracheal epithelial cell line 9HTEo [213]. In mice, TLR4 has been shown to recognize the F protein and activate NF-κB during RSV infection [203,214]. Accordingly, TLR4-deficient animals exhibit impaired NK cell function and increased viral load [205,215]. Defective TLR4 signalling has also been linked to increased pathology in a study of pre-term infants [216]. An essential role for IL-12, rather than TLR4, in susceptibility to RSV has also been proposed [214]; however, significant differences in experimental design limit the comparison of these apparently discordant studies [217].

In human lung fibroblasts and epithelial cells, the formation of dsRNA during RSV replication can activate TLR3-mediated immune signaling, leading to the upregulation of the chemokines RANTES and IP-10 [218]. TLR3-deficient mice have a predominantly Th2 response to RSV characterized by increased airway eosinophilia, mucus hypersecretion and expression of IL-5 and IL-13 [219]. RIG-I-induced IFN-β expression during RSV infection was recently shown to trigger TLR3 activation, suggesting that TLR3 mediates a secondary immune signaling pathway [220]. Interestingly, while TLR3 is involved in chemokine expression it has no role in RSV viral clearance, which is primarily mediated by the TLR2/TLR6 heterodimer [218,219].

In summary, the emerging picture of allergic asthma suggests that the disease can be mediated or exacerbated in genetically predisposed individuals by infection. In some cases these infections may induce an inflammatory state that protects against asthma, while in others the infection may elicit an acute allergic response or bias the host towards a subsequent Th2 response (figure 1).

## Conclusion

Innate immunity is a principal mechanism for the maintenance of lung tissue homeostasis despite continuous exposure to environmental irritants and potentially pathogenic microorganisms. In recent years tremendous progress has been made with regard to how the TLRs contribute to host defence and tissue repair. The insights that have arisen from this work allow one to postulate a few general principles with regard to lung innate immunity. First, acute pulmonary diseases such as ALI and bronchiolitis frequently develop into chronic inflammatory states (fibroproliferative ARDS) or exhibit a relapsing and remitting pattern (asthma). Second, infectious diseases are principal causes of sustained lung inflammation, as exemplified by severe influenza pneumonia that progresses to ARDS or severe RSV infection that precedes the development of asthma. Third, defective innate immunity contributes to the development of chronic obstructive lung diseases while directly or indirectly predisposing the host to infection, as observed in CF patients with chronic *P. aeruginosa *infection or acute exacerbations of COPD caused by *S. pneumoniae*. Finally, tissue repair and remodelling are crucial to the pathogenesis of lung inflammation as well as to host defense, and based on current data it appears that TLR-dependent mechanisms mediate the development of both processes.

Despite extensive research, many questions remain unanswered, including the relative contributions of TLR and non-TLR PRRs to lung inflammation and protective immunity, the precise nature of gene-environment interactions in asthma pathogenesis, the molecular mechanisms that negatively regulate the innate immune response during ALI, the failure of innate immunity to sterilize the lower respiratory tract in CF, and the role of innate immunity in tissue remodelling in asthma and COPD. A deeper understanding of the basic biology of TLRs will provide additional opportunities to elucidate the links between innate immunity and the development of acute and chronic inflammatory or infectious lung diseases. Ultimately, it is our hope that such knowledge will provide new strategies to limit the burden of human suffering and death due to respiratory disease.

## Competing interests

The authors declare that they have no competing interests.

## Authors' contributions

E.I.L., S.T.Q., and M.S. wrote the manuscript and approved the final text.
